# Low-Coherence Reflectometry for Refractive Index Measurements of Cells in Micro-Capillaries

**DOI:** 10.3390/s16101670

**Published:** 2016-10-11

**Authors:** Francesca Carpignano, Giulia Rigamonti, Giuliano Mazzini, Sabina Merlo

**Affiliations:** 1 Dipartimento di Ingegneria Industriale e dell’Informazione, Università degli Studi di Pavia, Via Ferrata 5, I-27100 Pavia, Italy; carpignano@unipv.it (F.C.); giulia.rigamonti01@universitadiapavia.it (G.R.); 2 Istituto di Genetica Molecolare IGM-C.N.R., Via Abbiategrasso 207, I-27100 Pavia, Italy; mazzi@igm.cnr.it; 3 Dipartimento di Biologia e Biotecnologie “L. Spallanzani”, Università degli Studi di Pavia, Via Ferrata 9, I-27100 Pavia, Italy

**Keywords:** low-coherence reflectometry, rectangular micro-capillary, infrared radiation, cell refractive index, cell cultures, drug discovery

## Abstract

The refractive index of cells provides insights into their composition, organization and function. Moreover, a good knowledge of the cell refractive index would allow an improvement of optical cytometric and diagnostic systems. Although interferometric techniques undoubtedly represent a good solution for quantifying optical path variation, obtaining the refractive index of a population of cells non-invasively remains challenging because of the variability in the geometrical thickness of the sample. In this paper, we demonstrate the use of infrared low-coherence reflectometry for non-invasively quantifying the average refractive index of cell populations gently confined in rectangular glass micro-capillaries. A suspension of human red blood cells in plasma is tested as a reference. As a use example, we apply this technique to estimate the average refractive index of cell populations belonging to epithelial and hematological families.

## 1. Introduction

In the past years, there has been a growing interest in the development of new methods for evaluating the refractive index (RI) of cells [1]. Knowing cell or tissue RI allows the improvement of the setup of diagnostic systems based on optical techniques, such as optical coherence tomography [2]. Cell RI has gained increasing attention also because it is related to biophysical cell properties and its value provides important information in chemical analyses, in studies on cell permeability and in hematology [3,4,5]. As an example, since hemoglobin is the main constituent of red blood cells (RBCs), an abnormal hemoglobin content, such as in iron-deficiency anemia or thalassemia, can induce an RI variation in RBCs [6]. Cell RI provides insights relative to the chemical composition and organization of the cell content and, therefore, this parameter has been considered highly significant also in cell biology and cyto-pathology [7,8,9]. Indeed, variations in tissue RI owing to changes in the cellular components play an important role in tissue light scattering effects, which have the potential to provide information about tissue pathology [10]. Moreover, in cancer biology, the RI is considered an indicator of cell malignancy [11,12] since the RI of proliferating cells is relatively higher than that of normal quiescent cells [7,11,13,14], probably because of a higher DNA content of the nucleus [15]. The RI is modulated by morphological and functional variations in the cell, particularly in transitions related to the loss of viability. Cell structural modifications induced by cell death (for apoptosis or for necrosis) are characterized not only by biochemical events but also by significant volume changes. The apoptotic pathway is associated with a cell shrinkage whereas necrosis induces an increase in cell size. These variations are currently detected by means of light scattering measurements in flow cytometry: forward scattering (FSC) refers mainly to cell size while side scattering (SSC) is related to the cell surface and to the inner structure of the cytoplasm and nucleus. FSC is strongly linked to RI since a cell volume increase/decrease induces an opposite variation of this parameter. RI may become a significant and intrinsic indicator of apoptotic cell status that is usually monitored via fluorescent detection of FITC-labeled Annexin-V [16]. A large variety of biomedical applications (among others, the area of drug discovery) may benefit from an alternative approach to scattering detection by flow cytometry (complex and expensive method) and to fluorescence measurements (characterized by a grade of cell toxicity) [17]. Several methods have been proposed to measure the cell RI, based on microscopy techniques [7,18,19,20,21,22,23,24], on optical trapping [25,26], or on hydro-mechanic holders [4]. Microscopy approaches require bulky imaging systems, complex analytical algorithms and allow measurements on living cells adherent to a slide or on histological slices [14]. Optical trapping techniques, on the other hand, require cell confinement by means of a light beam with high optical power, and thus may be potentially invasive for cells. Several optical interferometric configurations [27] have been developed such as micro-chip refractometers based on a Fabry-Pèrot resonant cavity [4,9]. Lue et al. have confined live cells in micro-channels of fixed vertical height, but accuracy has been limited by thickness variations in the horizontal plane [28]. Zilbershtein et al. [29,30] have demonstrated that Fourier transform infrared (IR) surface plasmon resonance can estimate the average refractive index of cell populations in view of its application to detect in real time the response of cells to environmental changes. Most of the reported approaches allow measurements on individual cells, even in three-dimensional (3D) [31], and on a relatively small number of them (up to a few hundreds). Although these methods can give very accurate results, they require, in most of the cases, complex or invasive measuring techniques, often based on lengthy processes. Therefore, there is still the need to demonstrate more attractive systems to estimate cell RI (suitable also for un-fixed samples) based on miniaturized devices, better if low-cost and available off-the-shelf, easily interfaced with the selected optical readout approach.

It would be also worthy to recall that single cells belonging to the same line and resulting from the same experimental culture could be in different functional states (quiescent or proliferating) and, if proliferating, also in various phases of the cell cycle: thus they might have different macromolecular contents and RIs. Testing a cell population allows us to yield the average RI value typical of that population (for example of a tumor) overall. In biomedical analyses, it is interesting to obtain data relative to the cell population, for example for monitoring the changes in RI in response to various physical and biochemical stimuli or, in the case of the drug-discovery field where a large number of tests is required, on cell cultures.

Recently, we have developed an optical low-coherence reflectometer that uses low-power infrared radiation to detect the optical path among inner interfaces of different multilayer structures. In a previous work [32], we demonstrated the functionality of this system for detecting the optical distance between the interfaces of rectangular micro-capillaries, also in the presence of transparent and homogeneous fluids inside the channel. Since the geometrical depth of the inner channel is fixed, we were able to recover the group refractive index of homogeneous liquid solutions filling the channel by measuring the variation of the optical path induced by the tested fluids. Rectangular-section micro-capillaries are standard microfluidic devices, available in several dimensions, that can be easily exploited in various applications without requiring facilities for micro-fabrication technologies. Method accuracy was previously demonstrated by measuring the group refractive index of water that was found in accordance with literature data specific for the near-infrared [32]. We also applied the implemented IR reflectometer for detecting the optical distance between the interfaces of silicon micromachined devices, composed by non-transparent layers in the visible wavelength range [33], thus demonstrating that measurements in the optical path domain are suitable for testing a wide variety of materials. 

In the present work, we are showing that infrared low-coherence reflectometry based on a low-power readout beam is suitable for measuring the variation of the optical path induced in the glass micro-capillaries by non-homogeneous and diffusing fluids, such as cell suspensions. In particular, as a proof of principle, this technique is here reported for estimating the average group refractive index of human red blood cells in plasma, as a benchmark. Then, as a preliminary example of the application, the technique is exploited for estimating the average group refractive index of different types of nucleated cells that are gently confined between the glass walls of rectangular micro-capillaries with a 50-µm-deep inner channel.

## 2. Materials and Methods

### 2.1. Cell Models

Measurements were performed on human cells belonging to the normal and to the transformed (malignant) compartments. Cells were used as un-fixed suspensions in culture media or ethanol-fixed and suspended in distilled water. Normal human red blood cells from healthy volunteer donor were used as a model with optical features already documented in the literature.

### 2.2. Normal Cells

Huker (normal human keratinocytes) were a gift of Riccardo Vicini (Dipartimento di Biologia e Biotecnologia “Lazzaro Spallanzani”, Università degli Studi di Pavia, Pavia, Italy) and grown as monolayer in D-MEM (with glucose 4.5 g/L + 10% Fetal Bovine Serum (FBS) + penicillin and streptomycin 1%). Before reaching confluence, cells were trypsinized and harvested with the standard methodology and recovered as a cell pellet in 1 mL conic plastic tubes. Sample were than split in two aliquots (of about three million cells each one): one was re-suspended in the culture medium (in the follow indicated as Medium 1) and used as un-fixed sample; the other one was washed two times in PBS and fixed in cold (−20 °C) 70% ethanol (for 1 h), then washed and re-suspended in distilled water. THP-1 (normal human monocytes), also provided by Riccardo Vicini, were grown in suspension in RPMI 1640 (with 5 × 10^−5^ M 2-mercaptoethanol + FBS 10% + penicillin and streptomycin 1%). Two aliquots of cells were then prepared, one as un-fixed cells in culture medium (indicated as Medium 2) and the other one as ethanol fixed cells, as previously stated for Huker cells. Reagents were purchased from Sigma Aldrich, Biowest and Life Technologies (Milano, Italy).

### 2.3. Transformed Cells

RPMI-7951 (malignant metastatic melanoma) were a gift of Anna Ivana Scovassi (Istituto di Genetica Molecolare IGM-C.N.R., Pavia, Italy) and grown as monolayer in D-MEM supplemented with 10% Fetal Calf Serum (FCS), 4 mM glutamine, 2 mM Na pyruvate, 100 U/mL penicillin and 0.1 mg/mL streptomycin. Before confluence, cells were treated as described above for Huker cells and the two (un-fixed and fixed) aliquots were obtained. Transformed human lymphoblasts were a gift of Ennio Prosperi (Istituto di Genetica Molecolare IGM-C.N.R., Pavia, Italy) and were grown in suspension in RPMI 1640 medium supplemented with 10% FBS. Aliquots of fixed and un-fixed cells were prepared also in this case. Reagents were purchased from Sigma Aldrich, Biowest and Life Technologies (Milano, Italy). 

### 2.4. Red Blood Cells

Normal human red blood cells (RBCs) were obtained from peripheral blood of a healthy volunteer donor. Five mL of anti-coagulated (with sodium citrate) whole blood was spun down at a Relative Centrifugal Force (RCF) of 150 g for 15 min. Plasma was aspirated and discarded and also the buffy coat at the interface (constituted by white blood cells) was eliminated. A small aliquot of the remaining concentrated red blood cells was transferred at the bottom of a 1 mL plastic conic tube (as done for the other cell models). Reagents were purchased from Sigma Aldrich (Milano, Italy).

### 2.5. Cell Staining

Huker and THP-1 cells were used for a preliminary evaluation of the filling procedure, being the first one epithelial and, therefore, biologically committed to aggregate, whereas the second is characterized as the largest cells (10–15 µm) in normal blood. Un-fixed cells were stained directly in their culture medium with 1 µg/mL of a supravital dye HO33342 (HO) for 30 min at 37 °C. Fixed cells were stained with 10 µg/mL of Propidium Iodide (PI) for 30 min in distilled water. After staining, cells were spun down and recovered at the bottom of a 1 mL plastic conic tube. The supernatant fluid was carefully aspirated and discarded, leaving a layer of liquid over the cell pellet. Red blood cells (used only as un-fixed sample) did not require any fluorescent staining: hemoglobin allows direct observation by bright field microscopy. Reagents were purchased from Sigma Aldrich (Milano, Italy).

### 2.6. Capillary Preparation

**Figure 1:**
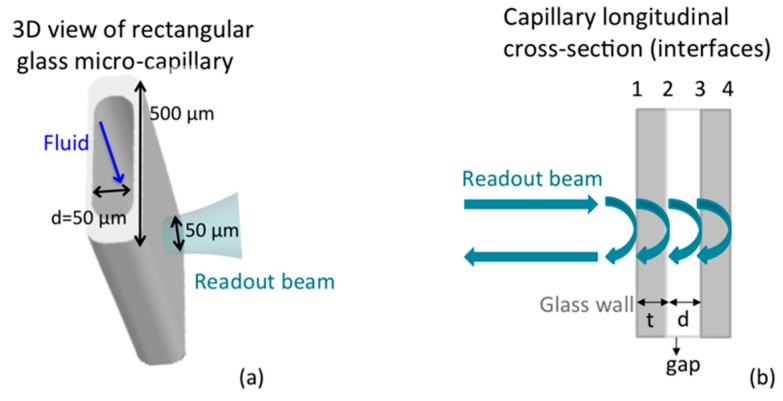
(**a**) 3D sketch of the glass rectangular micro-capillary; (**b**) Schema of a capillary longitudinal cross-section. The four interfaces of the capillary are highlighted.

Glass micro-capillaries with rectangular cross-section, also known as rectangle hollow capillary tubes, were purchased from Vitrotubes™ (VitroCom, Mountain Lakes, NJ, USA) and we tested their optical characteristics as reported in previous works [32]. The thickness of the front and back glass walls and the depth (d) of the inner channel were nominally equal to 50 μm whereas the width of the flat side was 500 μm (see Figure 1a).

The capillaries were 50 mm long. Standard tolerances of inner dimensions are of ±10%. The micro-capillaries were filled with cells (both un-fixed and fixed) simply by capillary forces. Just by dipping one side of the capillary for a few seconds in the bottom part of the conic tube (where they were temporarily stored, as explained in Section 2.5), the cell suspension filled up almost completely the capillary channel. Rectangular micro-capillaries were already demonstrated suitable for refractive index measurements of homogeneous fluids by means of the same instrumental configuration, applied also here, for implementing low-coherence reflectometry [32]. For a preliminary evaluation, fluorescence microscopy analyses of the filled capillaries were performed after cells were labeled with proper fluorescent dyes, except in the case of RBCs. As these cells are intrinsically red, they were observed and monitored by means of normal bright field setting of the same microscope. After filling, micro-capillaries were sealed on both sides with the mounting media “Moviol” in order to prevent any liquid evaporation and, therefore, ensure stability of the filler for at least a few hours. Finally, they were stored in vertical position for 1 h in order to guarantee the best cell compactness inside the channel. The micro-capillaries could be then gently manipulated without affecting the cell distribution inside the channel.

### 2.7. Microscopy Analyses of Cell Distribution Inside the Capillary

To evaluate the cell distribution (and behavior over the time) inside the capillaries, they were deposed on a normal glass slide and observed (with 4× up to 20× magnification) by means of a BX51 Olympus microscope (Olympus GmbH, Hamburg, Germany) equipped with a standard epifluorescence system. Up-right illumination was performed by a Mercury arc lamp (Osram HBO 100/2, Osram GmbH, Augsburg, Germany). For best HO observation, the excitation band was selected by a Band Pass Filter (BP366) and reflected by a dichroic mirror (DM 400), whereas the emitted fluorescence was selected by means of a barrier Long Pass Filter (LP) 450 nm. For best PI observation, the green excitation band was selected by an Interference Band Pass Filter (BP 530–560 nm) and reflected by a dichroic mirror (DM 590), whereas the emitted red fluorescence was observed thanks to a barrier Long Pass Filter (LP) 620 nm. Capillaries with RBCs were observed and monitored by means of the same microscope operated in a standard bright field mode. Sample images were taken at various magnifications with the Olympus Camedia C-4040 digital camera.

### 2.8. Instrumental Configuration

**Figure 2:**
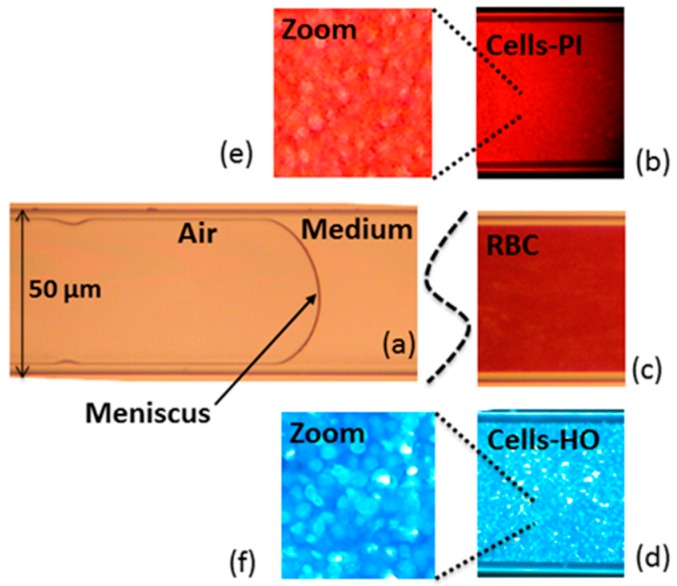
Microscopy images taken on capillaries after sample preparation. On the left, (**a**) typical image in bright field of the reference regions filled with air and medium separated by a meniscus. On the right, typical results obtained on regions with cells: (**b**) fluorescence microscopy image of fixed cells labeled with PI; (**c**) bright field microscopy image of RBCs; (**d**) fluorescence microscopy image of un-fixed cells labeled with HO; (**e**) zoom of (**b**) and (**f**) zoom of (**d**) for a better visualization of cells.

The optical setup was reported in detail in [32,33]. The all-fiber configuration was based on a Michelson interferometer with two bidirectional couplers with 50:50 splitting ratio and flat spectral response. Broadband radiation, after crossing the two couplers, was launched partly toward the capillary (“measuring arm”) and partly toward a reference translating-mirror (“reference arm”). Light spots with 50 µm diameter were generated on the targets at a working distance of 2.3 cm, resulting in a total investigated volume of about 100 pl. Radiation reflected by the mirror and the capillary was coupled back into the fibers and carried toward the InGaAs photodiodes incorporated in a balanced receiver. Interferometric fringes were recorded through an analog to digital (A/D) conversion board at a sampling frequency of 24 kHz with a personal computer. Figure 1a illustrates a sketch of the glass micro-capillary; as shown in Figure 1b, at each interface light is partly reflected and partly transmitted and the output backward signal is the result of the interference between the fields reflected from the capillary interfaces and from the reference mirror. For low-coherence reflectometers, the interferometric signal is generated only when the targets on both arms are at the same optical distance. As readout source we used a Tungsten lamp that provided a power spectral density of about −60 dBm/10 nm in the wavelength range from 1.2 μm to 1.7 μm, ensuring an axial resolution better than 3 μm [32]. All the experiments were carried on at room temperature, without a specific temperature control of the sample. Before performing reflectometric experiments, we analyzed (as explained in Section 2.6 and Section 2.7) the capillary samples to verify distribution and stability of the biological material filling the channel, by means of bright field and fluorescence microscopy. As an example, Figure 2 shows the microscopy images of capillary samples: it is possible to distinguish between regions with air, medium or cells.

## 3. Results

**Figure 3:**
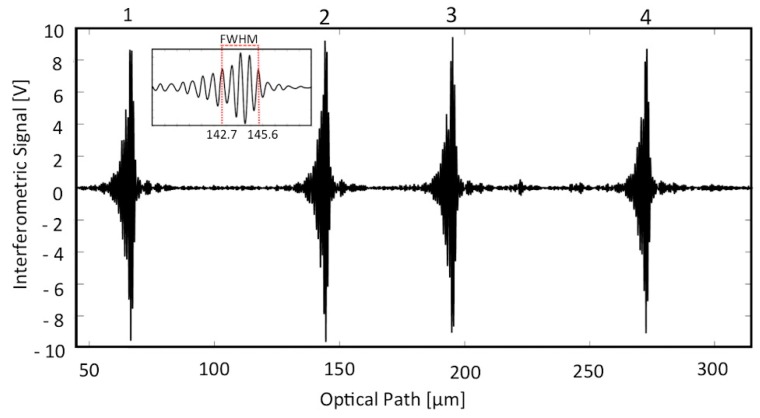
Interferometric signal as a function of the optical path obtained on 50 μm capillary in presence of air. 1, 2, 3, 4: interface positions, defined in Figure 1b. Inset: zoom of the second group of fringes. FWHM: Full width at half maximum.

**Figure 4:**
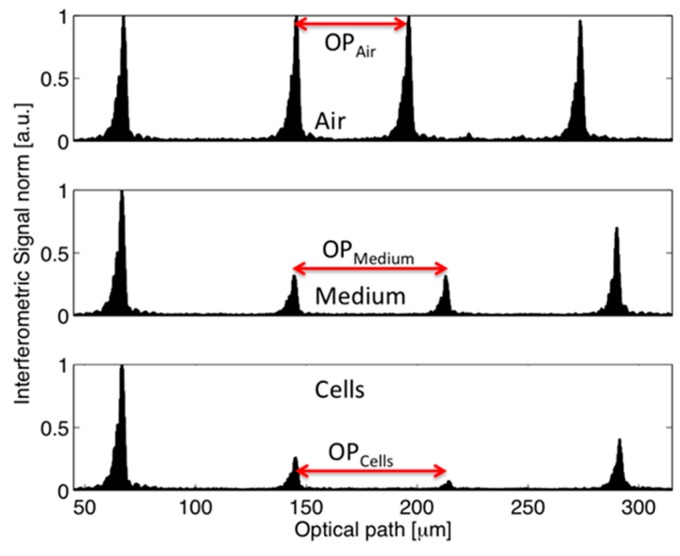
Normalized interferometric signal as a function of the optical path obtained on a 50-μm-deep capillary in presence of air (upper trace), culture medium (middle trace) and cells (lower trace). OP_Air_: optical path length of the channel with air; OP_Medium_: optical path length of the channel filled with culture medium; OP_Cells_: optical path length of the channel filled with cells.

The borosilicate glass capillaries with rectangular-section channels were used as micro-opto-fluidic devices for gently confining the cells under test conditions. The microscopy analyses demonstrated the presence of regions (also visible to the naked eye) with different fillings: cells, suspending medium (that was the culture medium for un-fixed cells, water for fixed cells and plasma for RBCs), and air, as shown in Figure 2. We thus performed low-coherence reflectometry in various positions along the capillary to obtain the optical path length of the empty channel (air filling), of the channel filled with medium and of the channel filled with cells. Whereas the interferometric signal was acquired in the time domain, the optical path (OP) was obtained by multiplying the travelling time T by the stage velocity v, i.e., OP = v · T, and v = 5 µm/s. The signal-to-noise ratio of the narrowband photodetected signal was greatly improved using a digital narrow-band filter. Figure 3 shows the filtered interferometric signal collected on a 50 μm capillary in the position corresponding to air. Figure 4 presents the absolute value (normalized to the peak) of that signal (upper trace) and of the signals collected in positions of the capillary where radiation crosses only the culture medium (middle trace) and where radiation crosses the cell suspension (lower trace).

**Figure 5:**
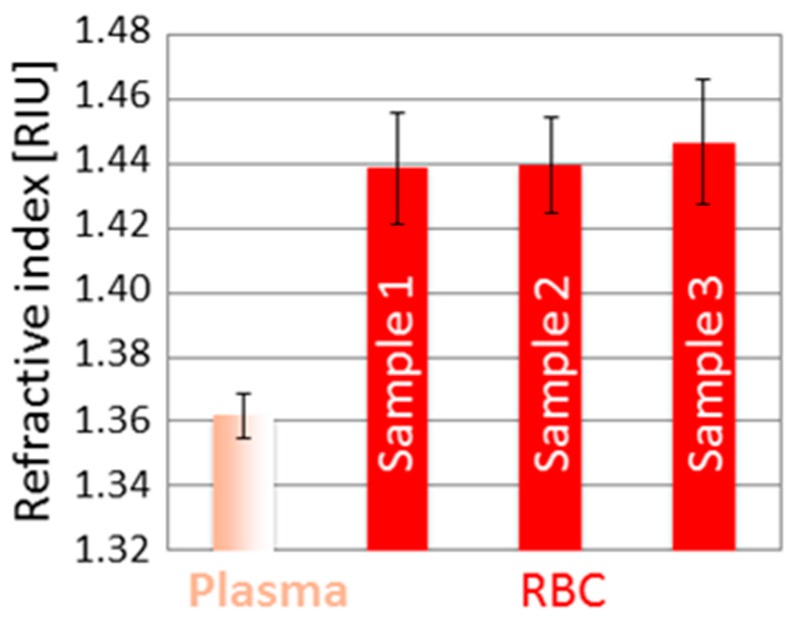
Column bar graph with average and standard deviation values of refractive index obtained for plasma and RBCs from three different samples.

The graphs in Figure 3 and Figure 4 show four groups of fringes: they correspond to the various interfaces crossed by the readout radiation, as numbered in Figure 1b. We notice that the optical path between the first and second group of fringes, calculated as the distance between their peak values, is constant because the presence of any kind of fluid does not affect the glass wall thickness. By analyzing the interferometric signals collected at a position of the capillary where the channel contains any kind of fluid (homogenous such as water, culture medium or plasma, but also non-homogeneous such as cellular suspension) different from air, an increase of the optical path between the second and third group is recorded, with respect to an empty capillary, as a results of the higher refractive index of the filling fluid. This experimental observation follows the theoretical relationship OP = n_g_ · d, where OP is the optical path between two consecutive interfaces, n_g_ is the group refractive index of the material confined between the considered interfaces and d is the geometrical distance between the same interfaces. From the relationship OP = n_g_ · d, since d = 50 µm (channel thickness), we get a responsivity *R* = ΔOP/Δn = 50 µm/RIU. As the relative position of the peak of the fringe group can be determined with a resolution better than ± λ/16 = ± 1.550 µm/16 = ± 0.097 µm, then Δn = ΔOP/*R* = ± 0.002 RIU, a suitable value for our studies. The stage velocity of 5 µm/s allows a 0.097 µm displacement to be performed in 0.02 s, corresponding to 480 data samples with a 24 kHz sampling frequency. The peak amplitude of the second and third group of fringes in the presence of any fluids is smaller than that of the first (and often also of the fourth) group because the refractive index difference between air and glass is greater than that between fluid and glass. The peak amplitude (normalized to the peak amplitude of first fringe group) of the second fringe group detected in the presence of cells inside the channel is smaller than the peak amplitude of the same group measured in the presence of culture medium because the refractive index of cells is greater than that of the culture medium. It should be observed that the peak amplitudes of the third and fourth fringe groups depend also on scattering and/or absorption properties of the filling fluid. Whereas fluid and cell absorption can be neglected in the near-infrared, scattering losses in the dense cell solution can play an important role in reducing the amplitude of the third and fourth peaks. The experimental values of the group RI of the various tested fluids and cell populations were then obtained as n_g,fluid_ = OP_fluid_/OP_Air_ (thus, n_g,Medium_ = OP_Medium_/OP_Air_ and n_g,Cells_ = OP_Cells_/OP_Air_). In all cases, we calculated the average and standard deviation on three repeated measures, also performed on different capillary samples. Figure 5 shows the results relative to the experimental values of the group refractive index obtained for plasma (n_g,Plasma_ = 1.362 ± 0.007 RIU) and red blood cells (n_g,RBC_ = 1.442 ± 0.017 RIU). Moreover, the RI estimated for RBCs is in good agreement with values previously reported in the literature by other authors, though obtained with a more complex setup and larger sample volume. Similar values were, for example, reported in [34,35,36] with tomographic measurements based on index matching. These comparisons were performed to support and validate our technique for refractive index estimation of non-homogeneous fluids, such as cell suspensions, gently confined in a micro-capillary.

**Figure 6:**
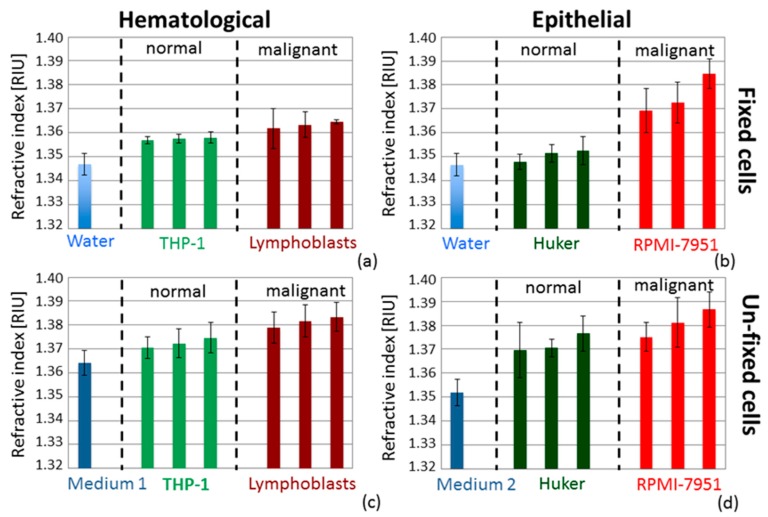
Column bar graphs with average and standard deviation values of refractive index obtained for four different cell lines and the medium in which they are suspended (i.e., water or Medium 1 and 2). Each graph compares RI values of normal and malignant cells with the same origin. (**a**,**b**) Fixed cells; (**c**,**d**) Un-fixed cells; (**a**,**c**) Hematological cells; (**b**,**d**) Epithelial cells.

In Figure 6 we present four graphs with the RI experimental values obtained on four different cell lines (two with epithelial origin and two with hematological origin) as well as on the suspending medium (i.e., water or Medium 1 and 2). Two graphs show the values obtained when considering fixed cells (a, b) and the others report the values obtained for un-fixed cells (c, d). These results clearly indicate that the refractive index of the cell suspension is always higher than that of the medium. A difference between the average refractive index of the samples containing normal and transformed (malignant) cells, also mentioned in the literature [13], was observed. We applied a one-sided Mann-Whitney test [37] to investigate the statistical significance of the difference and we obtained *p*-values < 0.05, in particular *p* = 0.04 × 10^−3^ for fixed Lymphoblasts versus THP-1 and *p* = 0.02 × 10^−3^ for fixed RPMI versus Huker; in the case of un-fixed cells, we calculated *p* = 0.04 × 10^−3^ for Lymphoblasts versus THP-1 and *p* = 19 × 10^−3^ for RPMI versus Huker. Despite the heterogeneity of the samples, the reported values seem to witness a statistical difference between the average RIs of transformed versus normal cells, even if it should be recognized that we are here reporting only preliminary results and the *p*-values were calculated on data ensembles of small size.

It is worthy to underline here that the arrangement of the cells inside the tested volume is random, so the measured optical path length does not correspond to a preferred axial direction of the cell. Cells are randomly oriented inside the tested volume as they are not grown adherent to the walls of the capillary.

## 4. Discussion

We have demonstrated the use of infrared low-coherence reflectometry to detect the average refractive index of non-homogeneous biological samples, such as cell suspensions, gently confined into rectangular glass micro-capillaries. The cells filled the channel just by capillary forces and we were able to detect the average refractive index of the analyzed cell population by means of the IR reflectometer. As a benchmark, we tested our system on red blood cells and we found results in substantial agreement with data reported in the literature. As an example, we applied the proposed combination of glass micro-capillary and low-power infrared reflectometry for estimating the average group refractive index of cell populations belonging to the epithelial and the hematological families. A difference was observed between the RI values estimated on samples of normal and malignant cells, but the result needs to be confirmed by further investigations. The proposed approach is simple and the measurements could be potentially performed with an automatic sequence, at several positions of the capillary, in order to have average data on a sufficiently large number of cells. This non-invasive method, based on a low-power, infrared readout beam, for measuring the group refractive index of non-homogeneous biological samples could be applied also on micro-fluidic systems, integrating micro-channels with rectangular cross-sections, allowing RI measurements on highly scattering biological samples [38]. As micro-capillaries can also be used as flow-through devices, it would in principle also be possible to detect in real time the response of a group of cells to environmental changes induced in the capillary itself. Biomedical research is often characterized by the requirement of sophisticated, complex and expensive instrumentation [39], that is sometimes unavailable in laboratories. On the other hand, routine applications in the same field will benefit from more compact and less expensive instrumental configurations, such as the one that we have proposed. This is the case for the example of marine biology (specifically in water pollution) where bacteria and algae may be differentiated on the basis of the refractive index [40,41]. A further advantage of this method consists of its applicability on fixed biological samples; thus, samples can be collected, stored and analyzed later on, even in a different location. Finally, another emerging area of future applications to be considered is the wide field of allergology where target cells, once activated by specific agents, immediately respond with macromolecular events located on the cell surface and shrinkage of the whole cell body. More specifically, the so-called “heavy metal” ions (and, among others, the Ni++) play a crucial role in the industry of cosmetics where a large number of specific tests are required to monitor the presence/absence of these metal ions; currently, the molecular steps are monitored by means of immuno-fluorescence cell labeling and flow cytometry. Evaluation of body size variations is usually performed by detecting FSC [16,42,43,44], and is thus an improvement in terms of reagent saving, but still time-consuming and costly. The close relation between FSC and RI [45] (detected also on fixed samples) will open new horizons in routine analyses in the drug discovery field. In conclusion, our technique provides an optical constant that is averaged over a population of about hundreds of cells. Whereas microscopy-based techniques can examine single cells in relatively small numbers, the method that we are proposing allows observation of the average behavior of a larger number of cells. It might be suitable for examining the changes in RI of a cell population to various physical and biochemical stimuli, as, for example, in the case of the drug discovery field where a large number of tests is required on cell cultures, or for sample pre-screening. Preliminary data presented in this manuscript represent a proof of principle of the method. Further refinements, such as implementation of better temperature and actuation controls, are under investigation.
